# PD85 Are Current Payment Models For Integrated Care Promoting The Uptake of Medical Device Innovations?

**DOI:** 10.1017/S0266462324003350

**Published:** 2025-01-07

**Authors:** Paula Closa Granados, Marcelo Soto, Ismail Abbas, Albert Alonso, Laura Sampietro-Colom

## Abstract

**Introduction:**

Integrated care (IC) is a patient-centered approach to the provision of health care that overcomes the fragmentation in the delivery of services that often occurs in older people with multiple comorbidities. Medical device innovations could help tackle this and other challenges of health systems when facing the adoption of high quality IC. Nevertheless, this should be accompanied by the right innovation funding scheme to encourage adoption.

**Methods:**

A systematic search strategy to identify payment models for IC was performed in PubMed for the period from 2014 to 2023 using the International Foundation for Integrated Care Knowledge Tree tool. This was complemented with both a gray literature search in Google and a benchmark of payment models for innovative medicines. A scoping review was then performed identifying the following information: payment scheme; advantages and disadvantages for IC; inclusion of innovation; type of IC program or initiative described (sectors and providers); population (therapeutic area); country; and whether the information was conceptual or empirical.

**Results:**

According to the literature reviewed, five payment schemes used in IC were identified. However, they were rarely used in isolation; more often a mix of payment schemes was used. Based on conceptual and empirical articles, the combination of bundled payment, shared savings, and pay for performance (based on the quality of care delivered) seemed to be the most comprehensive payment scheme. However, references to inclusion of innovation were not mentioned even in this mixed system. Payment systems for innovative medicines were benchmarked for medical devices.

**Conclusions:**

A mixed system of bundled payment, shared savings, and pay for performance appears to be the most complete payment model for IC, since it counteracts the limitations of individual funding schemes. No evidence was found on how to incentivize medical device innovation in the context of IC delivery. However, inspiration from current payment schemes for innovative medicines can provide a starting point for innovative medical device schemes.

